# Supplementary material for: Effect of Light-Emitting Diodes and Ultraviolet Irradiation on the Soluble Sugar, Organic Acid, and Carotenoid Content of Postharvest Sweet Oranges (Citrus sinensis (L.) Osbeck)
Source: Molecules. 2019 Sep 22;24(19):3440. doi: 10.3390/molecules24193440 (PMC6803866; doi:10.3390/molecules24193440)
Supplement: Supplementary file 1 [file molecules-24-03440-s001.pdf]

molecules

MDPI

Article

Effect of Light-Emitting Diodes and Ultraviolet Irradiation on the Soluble Sugar, Organic Acid, and Carotenoid Content of Postharvest Sweet Oranges (*Citrus sinensis (L.)* Osbeck)

Linping Hu [1,†], Can Yang [1,†], Lina Zhang [1], Jing Feng [1] and Wanpeng Xi [1,2,*]

[1] College of Horticulture and Landscape Architecture, Southwest University, Chongqing 400716, China; 18875065318@163.com (L.H.); yangcan0929@hotmail.com (C.Y.); zhangln0923@163.com (L.Z.); 15520104087@163.com (J.F.)
[2] Key Laboratory of Horticulture Science for Southern Mountainous Regions, Ministry of Education, Chongqing 400715, China
* Correspondence: xwp1999@zju.edu.cn; Tel.: +86-23-68250483; Fax: +86-23-68251274
† These authors contributed equally to this work.

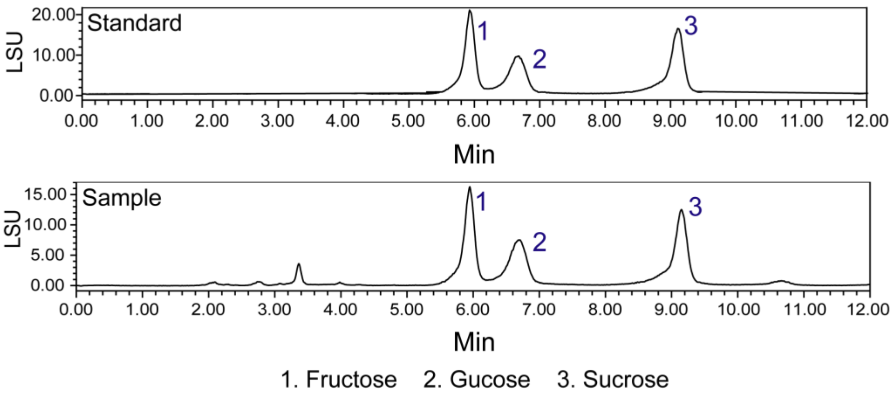

Figure S1. Representative high pressure liquid chromatography (HPLC) separation of soluble sugar standards and soluble sugars from the segment of postharvest fruit. Peaks: 1, fructose; 2, glucose; 3, sucrose.

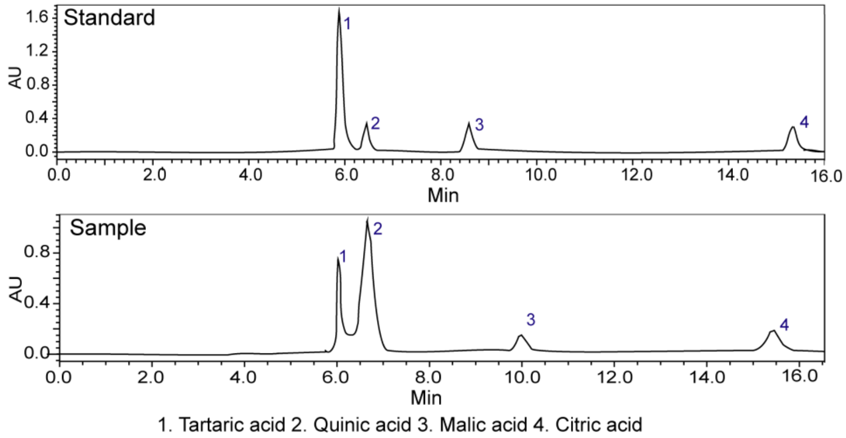

Figure S2. Representative high pressure liquid chromatography (HPLC) separation of organic acid standards and organic acids from the segment of postharvest fruit. Peaks: 1, tartaric acid; 2, quinic acid; 3, malic acid; 4, citric acid.

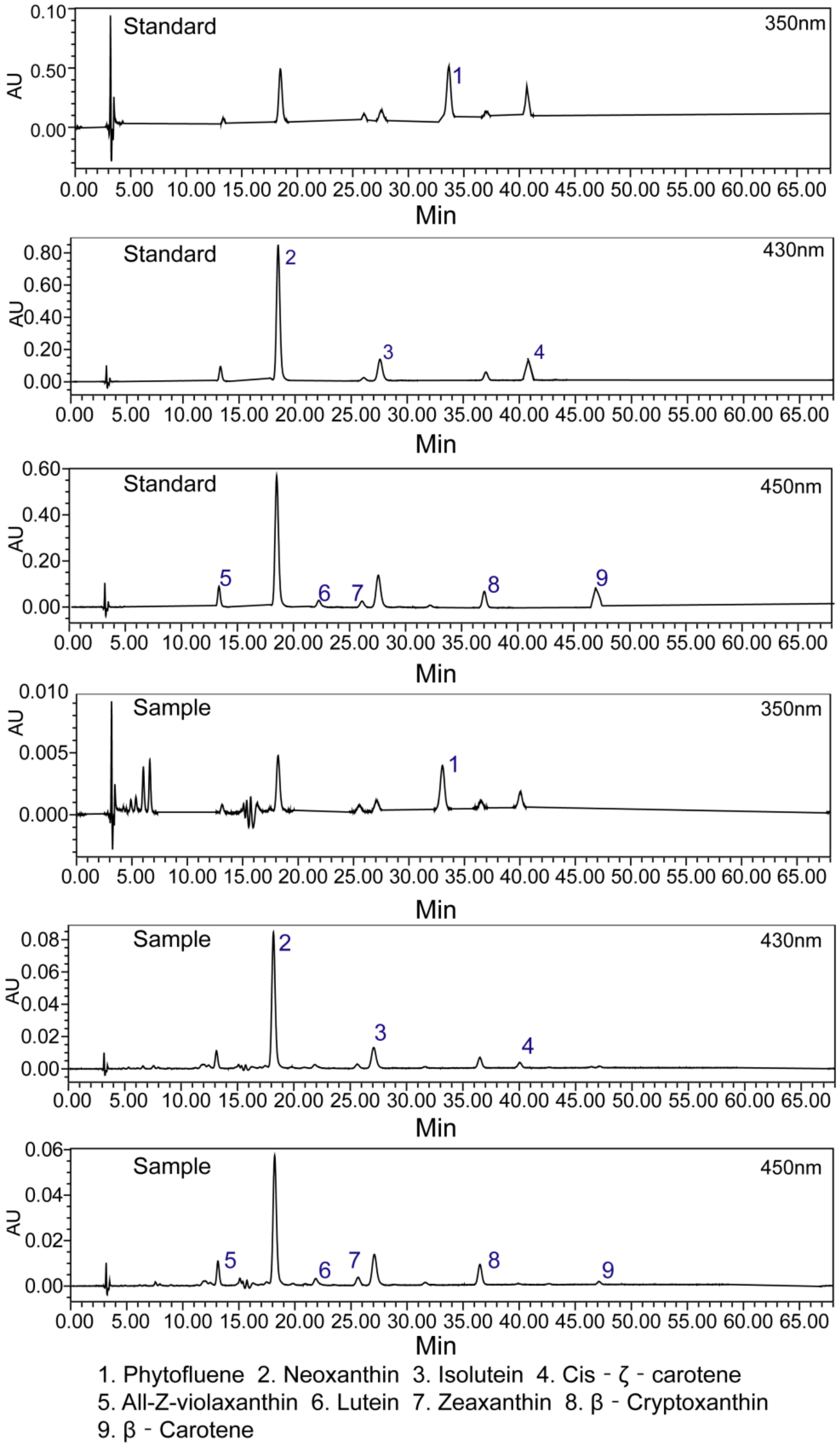

Figure S3. Representative high pressure liquid chromatography (HPLC) separation of carotenoid standards and carotenoids from the segment of postharvest fruit. Peaks: 1, phytofluene; 2, neoxanthin; 3, isolutein; 4, cis-ζ-carotene; 5, all-Z-violaxanthin; 6, lutein; 7, zeaxanthin; 8, β-cryptoxanthin; and 9, β-carotene.
